# Glucose Oxidase Biosensor Modeling and Predictors Optimization by Machine Learning Methods †

**DOI:** 10.3390/s16111483

**Published:** 2016-10-26

**Authors:** Felix F. Gonzalez-Navarro, Margarita Stilianova-Stoytcheva, Livier Renteria-Gutierrez, Lluís A. Belanche-Muñoz, Brenda L. Flores-Rios, Jorge E. Ibarra-Esquer

**Affiliations:** 1 Instituto de Ingeniería, Universidad Autónoma de Baja California, Mexicali, B.C. 21290, Mexico; margarita.stoytcheva@uabc.edu.mx (M.S.-S.); livier.renteria@uabc.edu.mx (L.R.-G.); brenda.flores@uabc.edu.mx (B.L.F.-R.); jorge.ibarra@uabc.edu.mx (J.E.I.-E.); 2 Computer Science Department, Universitat Politecnica de Catalunya, Barcelona 08034, Spain; belanche@lsi.upc.edu

**Keywords:** machine learning, biosensors, glucose-oxidase, neural networks, support vector machines, PLS, multivariate polynomial regression, optimization

## Abstract

Biosensors are small analytical devices incorporating a biological recognition element and a physico-chemical transducer to convert a biological signal into an electrical reading. Nowadays, their technological appeal resides in their fast performance, high sensitivity and continuous measuring capabilities; however, a full understanding is still under research. This paper aims to contribute to this growing field of biotechnology, with a focus on Glucose-Oxidase Biosensor (GOB) modeling through statistical learning methods from a regression perspective. We model the amperometric response of a GOB with dependent variables under different conditions, such as temperature, benzoquinone, pH and glucose concentrations, by means of several machine learning algorithms. Since the sensitivity of a GOB response is strongly related to these dependent variables, their interactions should be optimized to maximize the output signal, for which a genetic algorithm and simulated annealing are used. We report a model that shows a good generalization error and is consistent with the optimization.

## 1. Introduction

**Figure 1:**
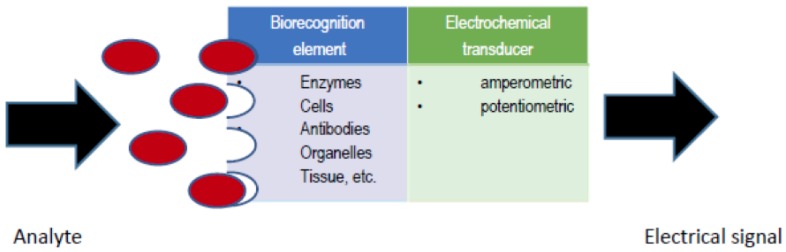
Electrochemical biosensor: the analyte is recognized by the bioreceptor followed by detection by the transducer, producing a measurable electric signal.

An electrochemical biosensor (see Figure 1) is an analytical device, which contains a biological recognition element in direct spatial contact with an electrochemical transducer, to obtain a measurable analytically-useful electrical signal by coupling biochemical and electrochemical events [1]. A number of variables affect the response of an electrochemical biosensor and therefore the biosensor analytical performance, including electrode variables (material, area and geometry), electrical variables (voltage, current, charge, impedance), electrolyte variables (bulk concentration, pH, solvent), reaction variables (kinetic and thermodynamic parameters) and external variables (temperature, pressure and time) [2]. Taking into account that the electrochemical biosensor response results from the overall interactions among dependent variables, statistical modeling is an efficient tool to predict, describe and optimize the electrochemical biosensor response and its analytical characteristics.

Most of the mathematical approaches have aimed at modeling the sensor response as a function of the kinetic behavior of the electrochemical biosensors. For example, Blaedel et al. [3] developed the first noteworthy model describing quantitatively the kinetic behavior of simple idealized enzyme sensors. It was applied to the treatment of the potentiometric response of urease electrodes. Using digital simulation, Mell and Maloy [4] modeled the steady-state current response of the amperometric stationary enzyme electrodes. Nevertheless, the lack of exactly defined boundary conditions to describe the mass transport restrains its application. Taking advantage of the well-known diffusion behavior of the rotating disk electrode, Shu and Wilson [5] demonstrated that the steady-state current response of the resultant amperometric enzyme sensor is in accordance, under mass transport limiting conditions at low substrate concentrations, with the Levich equation for the rotating disk electrodes [6]. Under catalysis-controlled conditions, the transfer function of the biosensor complies with the Michaelis-Menten equation [7].

Other techniques include methods to model the biosensor response by the analytical solution of partial differential equations, applicable to simple biocatalytic processes, as well as digital modeling using complex biocatalytical conversions, multi-part transducers’ geometry, and biocatalytic membrane structure; these are extensively reviewed by Baronas et al. [8] and Bartlett et al. [9]. More recently, Rangelova et al. [10] and Alonso et al. [11] demonstrated the potential of the Artificial Neural Networks (ANN) approach to electrochemical biosensor response modeling. Another promising method for analyzing overlapped signals, which cannot be calibrated and modeled by linear expressions, seems to be that of Support Vector Machines (SVM), displaying in general comparable to or better performance than ANNs and other statistical models [12].

The principle of the operation of the first-, second- and third-generation electrochemical biosensors for glucose determination, as well as their analytical performances, advantages and drawbacks are comprehensively described in the literature; see, e.g., [13,14,15,16,17]. The second generation amperometric GOB is well suited for blood glucose determination, since the oxygen dependence and the interference of the other components of the biological fluids are avoided. The challenges ahead rely on the development of biosensors with improved characteristics and optimized response for continuous glucose monitoring and point-of-care testing to better control and manage diabetes mellitus [13,14,15,18].

In this work, statistical Machine Learning (ML) regression models were chosen and applied as powerful techniques for estimating the current response of an amperometric second-generation glucose-oxidase biosensor (GOB), using p-benzoquinone as the electron-transfer mediator. Its function is based on the following enzymatic and electrochemical reactions:

$$\beta -D-\mathrm{Glucose}+GOD_{ox}\to D-\mathrm{Glucose}\mathrm{acid}+GOD_{red}$$

$$GOD_{red}+2M_{ox}\to GOD_{ox}+2M_{red}$$

$$2M_{red}\to 2M_{ox}+2e^{-}$$

where $GOD_{red}$ and $GOD_{ox}$ are the reduced and oxidized forms of the enzyme glucose-oxidase and $M_{red}$ and $M_{ox}$ are the reduced and oxidized forms of the mediator. The analytical signal is the current of $M_{red}$ oxidation, which is proportional to the glucose concentration.

## 2. ML Models for Regression

Let *Y* denote the response (or target) variable and $f(x_{1},x_{2},\ldots ,x_{p})$ the underlying *p*-variate function that represents the interaction between the predictors. The statistical Machine Learning (ML) perspective substitutes the *f* function by predefined algorithms with configurable parameters, leaving aside the task to establish complex mathematical relationships. The field offers a wide spectrum of regression models, which can be cast in some specific areas:

- Parametric linear models attempt to find a function defined by:
  (1) $$\hat{f}\left(x\right)=\beta_{0}+\sum_{j=1}^{p}x_{j}\beta_{j}$$
  where the objective is to find the *β* coefficients (or parameters) by means of some optimization criterion.
- Parametric non-linear regression finds a function that is a non-linear combination of the model parameters. For example, for p=1:
  (2) $$\hat{f}\left(x\right)=\beta_{0}+\beta_{1}x^{2}+\beta_{2}x^{3}+\beta_{3}\sin\left(\beta_{4}x\right)$$
- Semi-parametric regression, in which the predictor does not follow a predetermined form or definition; for example, a regression tree.

In the following sections, the chosen statistical regression models, intended to model the biosensor response in terms of some input variables, will be explained.

### 2.1. Partial Least Squares

Partial Least Squares (PLS) is considered a dimension reduction method, which identifies a new set of features $Z_{1},\ldots ,Z_{m}$ that are linear combinations of the original, then fits a linear model through least squares by using the *m* new features [19]. Let *X* be the inputs or predictors and $\hat{Y}$ the prediction; *X* and $\hat{Y}$ are decomposed into the following matrices:

(3) $$X=TP^{\prime }\;\mathrm{and}\;\hat{Y}=TBC^{\prime }$$

where *T* is the score matrix; P,C are the loadings (or weights) of X,Y, respectively; and *B* is a diagonal matrix. These new latent variables are sorted according to the amount of variance of $\hat{Y}$ that they explain, very much as in principal component analysis. Rewriting now $\hat{Y}$ as a regression model:

(4) $$\hat{Y}=TBC^{\prime }=XB_{PLS}$$

with

(5) $$B_{PLS}={\left(P^{\prime }\right)}^{†}BC^{\prime }$$

where ${\left(P^{\prime }\right)}^{†}$ is the Moore-Penrose pseudoinverse of $P^{\prime }$.

### 2.2. Artificial Neural Networks

**Figure 2:**
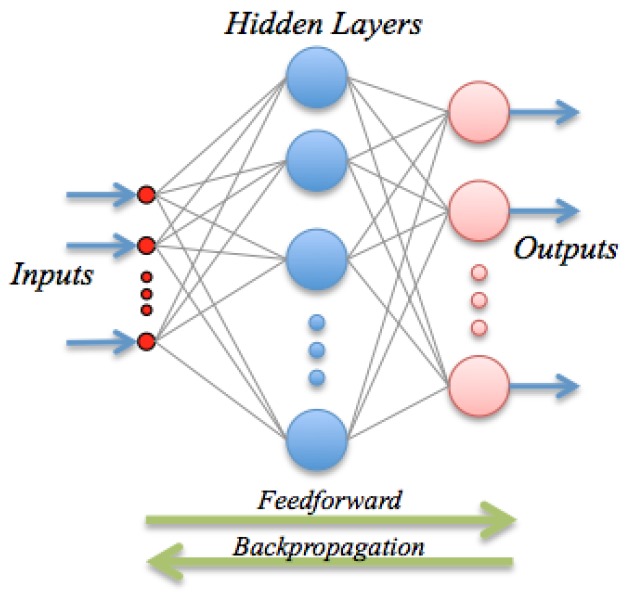
A simple graphical representation of a neural network.

ANN are inspired by the biological mechanism of brain and constitute an inspiration to develop mathematical representations of the information processing by neurons. They consist of processing units (nodes) interconnected through a certain topology; see Figure 2. The most widespread architecture is the feed-forward configuration, which assembles a linear combination of *m* fixed non-linear basis functions with parameters $\phi (\cdot ;v_{j})$ of the form:

(6) $$\hat{f}\left(x\right)=g\left(\sum_{j=1}^{m}w_{j}\phi \left(x;v_{j}\right)+b^{*}\right)$$

where *g* is a suitable non-linear activation function (e.g., a sigmoid), or the identity in the case of regression, and *w* is the vector of linear weights; the $v_{j}$ are the non-linear weight vectors, and $b^{*}$ is the bias or offset. The basis functions typically have the form:

(7) $$\phi \left(x;v_{j}\right)=g\left(\sum_{k=1}^{p}v_{jk}x_{k}+b_{j}\right)$$

Besides its formal definition, a neural network involves also the optimization procedure: once the output has been computed, the error, i.e., the difference between the predicted value and the observed value, is back-propagated. These error signals are then used to adjust the weights by a number of strategies; in this paper, we use a Levenberg-Marquardt method.

### 2.3. Support Vector Machines

The Support Vector Machine for Regression (SVMR) has become a popular tool for the modeling of non-linear regression tasks [20]. The SVMR is a nonlinear kernel-based method, which attempts to estimate a regression hyperplane $\hat{f}$ with a small risk in a high-dimensional feature space. Unlike the classical least-squares solution for linear fitting, SVMR tries to minimize the *ε*-insensitive loss function. This imposes a linear penalty when the value of the estimate $\hat{f}\left(x\right)$ with respect to the corresponding observed *y* is off-target by *ε* or more, as ${\left|\cdot \right|}_{\varepsilon }=\max\left\{0,|\cdot |-\varepsilon \right\}$, usually leading to sparser representations, entailing both algorithmic and representational advantages [21].

Let H be a real RKHS (Reproducing Kernel Hilbert Space) with kernel *κ*. The input data are transformed with a feature map Φ:X→H, to obtain the new dataset ${\left\{\left(\Phi \left(x_{i}\right),y_{i}\right)\right\}}_{i=1}^{N}$, where X is the input space. In an SVMR, the aim is to find a function $\hat{f}:{\langle \Phi \left(x\right),w\rangle }_{H}+b$, for some w∈H and b∈R, which is as flat as possible and deviates a maximum of *ε* from the given target values $y_{i}$, for all i=1,⋯,N.

The usual formulation of the optimization problem is as the dual of the convex quadratic program:

(8) $$\begin{array}{c} \begin{array}{cc} \min_{w\in H,b\in R} & \frac{1}{2}{∥w∥}_{H}^{2}+C\sum_{i=1}^{N}\left(\xi_{i}+\xi_{i}^{*}\right)\\ \mathrm{subject}\mathrm{to} & \left\{\begin{array}{ccc} y_{i}-{\langle \Phi \left(x_{i}\right),w\rangle }_{H}-b & \leq & \varepsilon +\xi_{i}\\ {\langle \Phi \left(x_{n}\right),w\rangle }_{H}+b-y_{i} & \leq & \varepsilon +\xi_{n}^{*}\\ \xi_{i},\xi_{i}^{*} & \geq & 0 \end{array}\right. \end{array} \end{array}$$

for i=1,⋯,N. To solve Equation (8), one considers the dual problem derived by the Lagrangian:

(9) $$\begin{array}{c} \begin{array}{cc} \max_{\alpha ,\alpha^{*}} & \left\{\begin{array}{c} -\frac{1}{2}\sum_{i,j=1}^{N}\left(\alpha_{i}-\alpha_{i}^{*}\right)\left(\alpha_{j}-\alpha_{j}^{*}\right)\kappa \left(x_{i},x_{j}\right)\\ -\varepsilon \sum_{i=1}^{N}\left(\alpha_{i}+\alpha_{i}^{*}\right)+\sum_{i=1}^{N}y_{i}\left(\alpha_{i}-\alpha_{i}^{*}\right) \end{array}\right.\\ \mathrm{subject}\mathrm{to} & \sum_{i=1}^{N}\left(\alpha_{i}-\alpha_{i}^{*}\right)=0\mathrm{and}\alpha_{i},\alpha_{i}^{*}\in \left[0,C\right] \end{array} \end{array}$$

Exploiting the saddle point conditions, it can be proven that $w=\sum_{i=1}^{N}\left(\alpha_{i}-\alpha_{i}^{*}\right)\Phi \left(x_{i}\right)$; given that $\kappa \left(x,x^{\prime }\right)={\langle \Phi \left(x\right),\Phi \left(x^{\prime }\right)\rangle }_{H}$, the solution becomes:

(10) $$\begin{array}{c} f\left(x\right)=\sum_{i=1}^{N}\left(\alpha_{i}-\alpha_{i}^{*}\right)\kappa \left(x_{i},x\right)+b,\;x\in X \end{array}$$

## 3. Experimental Work

### 3.1. Biosensor Data

The GOB used incorporates a p-benzoquinone-mediated amperometric graphite electrode with covalently-linked glucose-oxidase. The mediator is responsible for the electronic transfer between the enzyme and the electrode surface. Additionally, the following reagents were used: glucose-oxidase (E.C. 1.1.3.4. from *Aspergillus*, 1000 U/mg), *N*-cyclohexyl-*N*’-[2-(methylmorpholino)ethyl] carbodiimide-4-toluenesulfonate (Merk) and glucose. Amperometric data acquisition was achieved using a Radelkis OH-105 polarograph. The amperometric or electrical response was analyzed under different conditions of the glucose (in mmol/L), pH, temperature (in degrees Celsius) and concentration of the mediator, the p-benzoquinone (in mmol/L). Measured values for these input variables are described in Table 1.

The resulting data file consists of 320 rows (observations) and five columns: four predictive variables and a continuous target variable, which corresponds to the biosensor amperometric response, measured in mA. The predictive variables (glucose, p-benzoquinone, temperature and pH) are available for all combinations of input values in Table 1. All of the variables (except the GOB response) are standardized to zero mean, unit standard deviation. Finally, the data file is shuffled to avoid predefined ordering biases.

### 3.2. Experimental Settings

The dataset was randomly partitioned into a training set (70%) and a test set (30%). The training phase was conducted by 30 × 10 cross-validation (30 times of 10-fold cv). The true generalization capacity was assessed by evaluating the trained models in the test set. The performance measure used was the Normalized Root Mean Square Error (NRMSE):

(11) $$\sqrt{\frac{\sum_{i=1}^{N}{\left(\frac{e_{i}}{1-h_{ii}}\right)}^{2}}{\sum_{i=1}^{N}{\left(y_{i}-\bar{y}\right)}^{2}}}$$

where $e_{i}$ are the residuals and $h_{ii}$ the leverage of observation $x_{i}$. Three regression algorithms were used in the biosensor response prediction, the Partial Least Squares algorithm (PLS), a Support Vector Machine for Regression with Linear (SVMR-Lin) and Radial Basis Function (SVMR-RBF) kernels and an ANN for regression with the Levenberg-Marquardt backpropagation method. Optimal parameters were selected by grid search as follows: the SVMR complexity parameter *ε* and the RBF smoothing parameter *γ* were varied logarithmically in $2^{-6}\ldots 2^{6}$ and $10^{-1.5}\ldots 10^{1.5}$, respectively; for the ANN, the number of hidden layers was fixed to four neurons.

### 3.3. Hardware and Software

The models and experiments were coded in the MATLAB^®^ language, Version 2012a, and run on an Ubuntu Linux server, v. 11.10, with an Intel(R) Xeon(R) CPU E5620 @2.40 GHz and eight cores. The SVMR-RBF libraries were embedded into the MATLAB environment using the LIBSVM MATLAB^®^ Support Vector Machine Toolbox [22].

## 4. Results and Discussion

**Figure 3:**
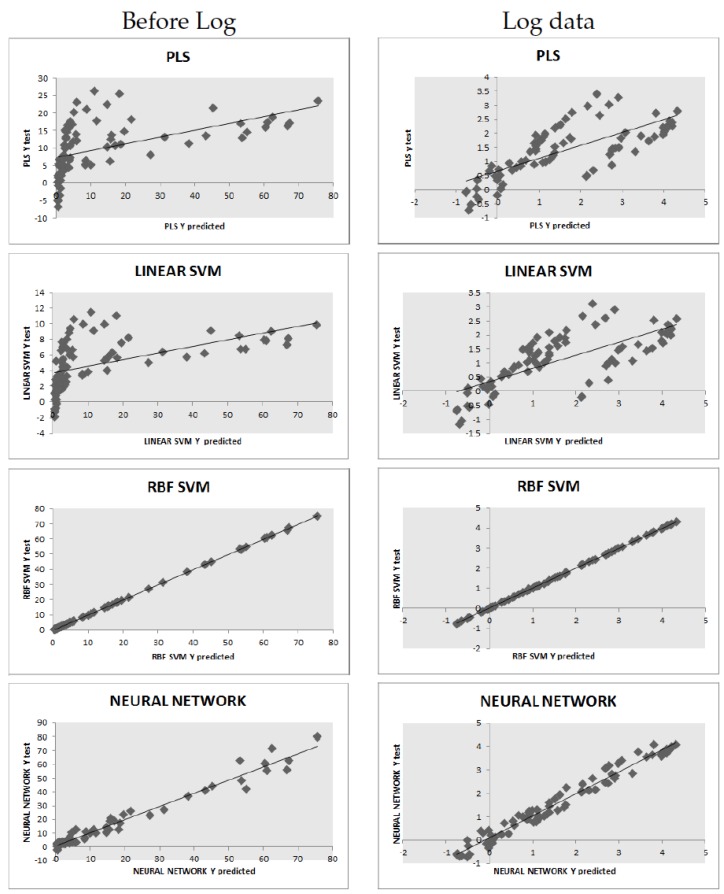
Regression plot for test data: observed target value vs. predicted target values, before and after taking the log the targets.

Two groups of experiments were performed, by training and testing the learning algorithms with and without applying the natural log to the target variable. Table 2 shows the cross-validation NRMSE for each learner, before log and log data, resp. It is clearly seen that the linear models, PLS and the linear SVM, are outperformed by the non-linear models, the ANN and the SVMR-RBF, the latter being the best model overall, although the difference of two models is not statistically significant: a Wilcoxon signed-rank test comparing the results, in the log data, shows that the null hypothesis that the difference between the cross-validation NRMSE medians is zero cannot be rejected at the 95% level (*p*-value =0.125). The test $R^{2}$ regression coefficients are also shown. For completeness, we also display the full test prediction plots for all models; see Figure 3.

**Figure 4:**
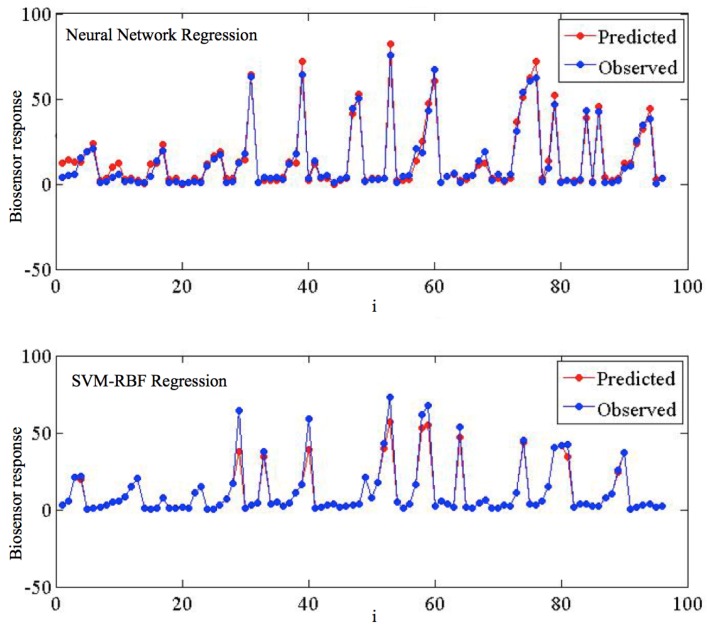
Detailed regression plot for the test data: observed and predicted values rendered by the ANN and the SVMR-RBF models on log data.

Figure 4 details the predicted vs. observed target values for the SVMR-RBF and the ANN. Despite most of the signal being very satisfactorily predicted, some of the points present divergences w.r.t. the observed target values; specifically, very high peaks are in general underestimated.

**Figure 5:**
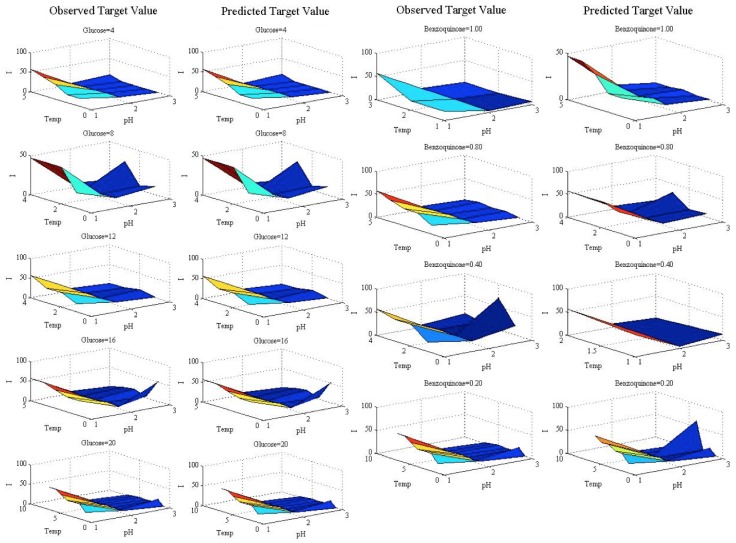
Observed vs. predicted target values with p-benzoquinone fixed to 0.2 (Columns 1–2) at different glucose values and glucose fixed to four at different p-benzoquinone values (Columns 3–4).

To explore this phenomenon, Figure 5 shows particular sections of the target value by displaying the observed and predicted values in the test set. The charts are formed by fixing the value of the benzoquinone to 0.2 for different glucose values, and by fixing the value of the glucose to four for different benzoquinone values. The *X*-*Y* axes are the temperature and pH variables, and the vertical axis shows the target value (biosensor response). The slight differences pointed out (for high-valued outputs) can be seen in Columns 3–4 of Figure 5.

### 4.1. Artificial Neural Network Model

**Figure 6:**
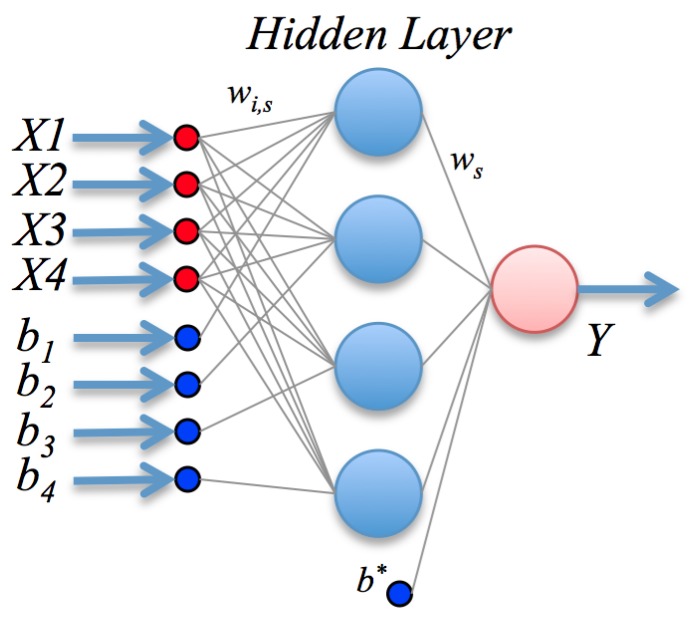
Artificial neural network final model configuration.

Letting $X=(X_{1},X_{2},X_{3},X_{4})$ represent the glucose, p-benzoquinone, temperature and pH and *Y* the biosensor response, the mathematical model found by the ANN, seen graphically in Figure 6, is assembled as (refer to the description in Section 2.2):

(12) $$\begin{array}{c} Y\left(X\right)=b^{*}+\sum_{j=1}^{m}w_{j}\phi \left(X;v_{j}\right) \end{array}$$

where m=4 and the basis functions use the hyperbolic tangent sigmoid function as *g*; the vector *w* of linear weights and bias $b^{*}$ are given by:

$$\begin{array}{c} \left(\begin{array}{c} w_{1}\\ w_{2}\\ w_{3}\\ w_{4}\\ b^{*} \end{array}\right)=\left(\begin{array}{c} -0.345672\\ -0.628801\\ 0.362671\\ -4.781217\\ -5.092845 \end{array}\right) \end{array}$$

The non-linear weight vectors $v_{j}$ and biases $b_{j}$ for each neuron *j* in the hidden layer are conveniently expressed in matrix form as:

$$\begin{array}{c} v_{j,k}=\left(\begin{array}{cccc} -13.292032 & -0.733296 & 0.643120 & -0.039301\\ -0.087084 & -0.215409 & 0.043895 & -0.007911\\ 133.065337 & -0.576762 & 4.903561 & 0.125645\\ 139.782513 & 5.381696 & 0.824023 & -4.086904 \end{array}\right) \end{array}$$

$$\begin{array}{c} b=\left(\begin{array}{c} -21.431381\\ 1.841679\\ 1.449347\\ -4.925324 \end{array}\right) \end{array}$$

### 4.2. Support Vector Machine RBF Model

The SVMR-RBF parameters that offer the best performance (lowest cross-validation NRMSE) are found to be as follows:

$$\begin{array}{c} \left(\begin{array}{c} C\\ \gamma \\ \varepsilon \end{array}\right)=\left(\begin{array}{c} 16\\ 5.6569\\ 0.95 \end{array}\right) \end{array}$$

The *C* and *ε* parameters are used in solving the optimization problem as described in Section 2.3; *γ* is used in the computation of the kernel function k(·,·), in our case the RBF kernel, defined as:

$$\kappa \left(x_{i},x_{j}\right)=\exp\left(-\gamma \;{∥x_{i}-x_{j}∥}^{2}\right)$$

## 5. Optimization of Experimental Conditions

The experimental results show very low prediction errors for GOB modeling through statistical learning methods from a regression perspective. Such models would be effective in case there is an interest in embedding them as a sub-system of other analytical processes.

These models are black boxes, in that they can be viewed solely in terms of their inputs and output, without any knowledge of the internal workings. However, a more in-depth theoretical modeling of the biosensor response would enable a better understanding about the importance of the factors affecting its analytical performance in terms of dynamic linear concentration range, sensitivity and the limit of detection, among others. Moreover, it would facilitate its optimization in a given matrix. Some current efforts are directed toward sensitivity improvement and lowering of the limit of detection by maximization of the biosensor response.

**Figure 7:**
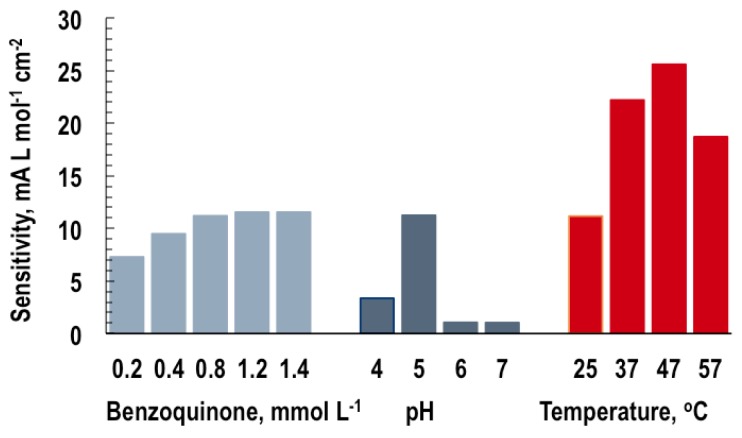
Biosensor sensitivity: dependence on the input variables, p-benzoquinone concentration, pH and temperature.

The sensitivity and linear concentration range of steady-state calibration curves are determined by plotting the steady-state responses (in our case, the GOB response), possibly corrected for a blank signal, vs. the analyte concentration (the glucose). The sensitivity is the slope of the calibration curve and is used for the evaluation of biosensor capabilities: a more sensitive device responds to smaller amounts or weaker signals. Thus, there is a need for analytical procedures for sensitivity maximization, i.e., the finding of input values that yield a maximum biosensor response. Since this is seen to strongly depend on the input variables, p-benzoquinone concentration, pH and temperature (see Figure 7), the aim of the analyses in this section was to determine the impact and optimum values of these individual input variables plus the glucose on the GOB response.

In this vein, the two developed regression models—the ANN and the SVMR-RBF—are excellent predictive models (aimed at generalization), but are not amendable to be optimized, in the sense of assessing the best input values to get the maximum output. Instead, polynomial optimization (of limited degree) is a more feasible task: once the coefficients are found, the polynomial can be optimized by a number of methods. The idea can be summarized in the following procedure:

1. Approximate a third-degree polynomial model by Ordinary Least Squares (OLS) estimation.
2. Maximize the polynomial as a function of the input variables.
3. Use these values to find the best output or biosensor response.

As in Section 4, letting $X=(X_{1},X_{2},X_{3},X_{4})$ represent the glucose, p-benzoquinone, temperature and pH and *Y* the biosensor response, a third order polynomial was assembled as follows:

(13) $$\begin{array}{ll} p(X)= & -2357.913\\ & -10.925X_{1}-41.378X_{2}-3.858X_{3}+1365.349X_{4}\\ & +0.578X_{1}X_{2}+0.232X_{1}X_{3}+3.790X_{1}X_{4}\\ & +0.764X_{2}X_{3}+19.766X_{2}X_{4}+0.639X_{3}X_{4}\\ & +0.066X_{1}X_{2}^{2}-0.002X_{1}X_{3}^{2}-0.398X_{1}X_{4}^{2}\\ & -0.014X_{1}^{2}X_{2}-0.001X_{1}^{2}X_{3}+0.0209X_{1}^{2}X_{4}\\ & -0.006X_{2}X_{3}^{2}-2.062X_{2}X_{4}^{2}-0.123X_{3}X_{4}^{2}\\ & -0.108X_{2}^{2}X_{3}+2.577X_{2}^{2}X_{4}+0.010X_{3}^{2}X_{4}\\ & -0.093X_{1}^{2}-31.360X_{2}^{2}+0.054X_{3}^{2}-255.292X_{4}^{2}\\ & +0.001X_{1}X_{2}X_{3}-0.0517X_{1}X_{2}X_{4}-0.006X_{1}X_{3}X_{4}-0.025X_{2}X_{3}X_{4}\\ & -0.001X_{1}^{3}+8.052X_{2}^{3}-0.001X_{3}^{3}+15.705X_{4}^{3} \end{array}$$

In order to maximize *p*, two well-known optimization algorithms were used: a genetic algorithm and simulated annealing, briefly described next. A Genetic Algorithm (GA) is a method for solving optimization problems based on natural selection, mimicking biological evolution by borrowing ideas from the dynamics of natural populations. It works under the assumption that the strongest individuals will prevail through time and, hence, their best features. Given a population, the algorithm iteratively selects individuals that represent the best characteristics of the population, as measured by a certain fitness function. These individuals are taken as a seed to produce the next generation by the use of genetic operators (crossover and mutation). At each step, the current population seeks to enhance the fitness function, eventually evolving to an optimal solution [23].

Simulated Annealing (SA) is a stochastic technique inspired by statistical mechanics for finding near globally optimum solutions to large (combinatorial) optimization problems. The algorithm works by assuming that some part of the current solution, i.e., the input variables assigned with some real value within their respective range, belongs to a potentially better one, and thus, this part should be retained by exploring neighbors of the current solution; SA has the ability to jump from valley to valley and to escape or simply avoid sub-optimal solutions [24].

Table 3 shows the results of this optimization process; it also shows the ANN and SVMR-RBF response when evaluated in the same solution, as a reference. Both the GA and SA show very similar values for the polynomial model, the small difference being attributable to rounding, which are achieved at the same readings for the inputs, a possible indication that a true maximum has been reached. Upon evaluation of the ANN and SVMR-RBF on these values, the results are again remarkably similar, a fact suggesting that the predictive models are essentially correct.

## 6. Conclusions and Future Work

Several statistical machine learning methods have been used to model the amperometric response of a GOB. The reported experimental results show a promising very low prediction error of the biosensor output by using artificial neural networks and support vector machines for regression. It has also been shown that the relationship between the available predictors (temperature, benzoquinone, pH and glucose concentration) and the response corresponds to a non-linear behavior. In biosensor response analysis, there is sometimes a need to find the optimum predictor values, namely those that yield a maximum response. It has been shown that a particular combination of the available predictors is able to deliver a maximized value for the predictive models. We thus recommend the learned SVMR-RBF model (with parameters C=16, γ=5.6569 and ε=0.95) as a solid predictor of the amperometric response.

Glucose monitoring by means of a GOB can constitute a valuable ally to diabetic patients. GOBs are solid candidates for fast, reliable and inexpensive monitoring, in order to avoid serious collateral chronic complications; however, their design is still under development, in order to improve both their predictive accuracy and stability upon changing conditions. In a biosensor design scenario, mathematical modeling is a highly promising tool, given that it facilitates computational simulations, avoiding destructive testing, as long as time and resources permit. In this sense, the experimental proposal and conditions offered in this paper could be applied for other scenarios in the wide spectrum of bio-sensing technology.

Future work will include the fine-tuning of the small divergences found in the prediction of peaks. One possible direction could be to model the signal as a wave, where it could be natural to use nonparametric or local regression models, such as splines or wavelets [25]. 

## Figures and Tables

**Table 1:** Input variables describing the Glucose-Oxidase Biosensor (GOB). Each column is the set of available values for each variable.

Glucose	pH	Temperature	p-Benzoquinone
(mmol/L)		(Celsius)	(mmol/L)
4	4	20	1
8	5	37	0.8
12	6	47	0.4
16	7	57	0.2
20	-	-	-

**Table 2:** Obtained cross-validation Normalized Root Mean Square Error (NRMSE) errors and test $R^{2}$ regression coefficients.

Regression Method	Before Log		Log Data	
	NRMSE	$R^{2}$	NRMSE	$R^{2}$
PLS	0.50	0.509	0.26	0.763
SVMR-Lin	1.44	0.520	0.28	0.718
SVMR-RBF	0.03	0.999	0.01	0.999
ANN	0.11	0.984	0.05	0.980

**Table 3:** Optimum values for the predictors found by a GA, SA and evaluation of the ANN and SVMR-RBF responses on these values. Biosensor output is in mA.

	GA	SA	ANN	SVMR-RBF
Max Output	57.86	58.01	58.10	57.96
Glucose	20	20	-	-
Benzoquinone	1	1	-	-
T	45	45	-	-
pH	5	5	-	-

## Supplementary Materials

Biosensor data set as described in Section 3.1 are available from the authors at http://nova.mxl.uabc.mx/~fernando/sensors16_data.
